# Airborne dimethyl sulfide (DMS) cues dimethylsulfoniopropionate (DMSP) increases in the intertidal green alga *Ulva fenestrata*

**DOI:** 10.1038/s41598-023-30881-9

**Published:** 2023-03-15

**Authors:** Kathryn L. Van Alstyne, Jennifer K. Butler, Neal Smith

**Affiliations:** Shannon Point Marine Center, Western Washington University, 1900 Shannon Point Road, Anacortes, WA 98221 USA

**Keywords:** Ecology, Marine biology, Plant ecology, Plant signalling, Chemical ecology

## Abstract

Although the use of airborne molecules as infochemicals is common in terrestrial plants, it has not been shown to occur in an ecologically relevant context in marine seaweeds. Like terrestrial plants, intertidal plants spend part of their lives emersed at low tide and release volatile organic compounds (VOCs) into the air when they are grazed or physiologically stressed. We hypothesized seaweeds could use airborne VOCs as infochemicals and respond to them by upregulating a keystone defensive metabolite, dimethylsulfoniopropionate (DMSP). We conducted laboratory and field experiments in which *Ulva fenestrata* was exposed to airborne dimethyl sulfide (DMS), a volatile antiherbivore and antioxidant metabolite released when the seaweed is grazed or physiologically stressed. In the laboratory, *U. fenestrata* exposed to DMS had 43–48% higher DMSP concentrations, relative to controls, 6–9 days after exposure. In the field, *U. fenestrata* 1 m downwind of DMS emitters had 19% higher DMSP concentrations than upwind seaweeds after 11 days. To our knowledge, this is the first demonstration of a marine plant using an airborne molecule released when damaged to elicit defensive responses. Our study suggests that the ability to detect airborne compounds has evolved multiple times or before the divergence of terrestrial plants and green algae.

## Introduction

Terrestrial plants use volatile organic compounds (VOCs) as airborne cues and signals to communicate and detect information about neighboring plants or plant tissues^1,2^. These chemical messengers can mediate interactions among organisms, for example, by signaling pollinators, seed dispersers, beneficial microbes, and predators^3^. They also can be detected by neighboring plants or tissues to gain information about the emitter's physiological condition or whether herbivores are attacking it. When used as indicators of herbivore attack, VOCs are released due to damage to the emitting individual, transported through the air, and detected by receptors on nearby plants or tissues. The receiver then upregulates the production of defensive metabolites to prevent or reduce damage from future attacks by grazers or primes tissues so that they can respond more quickly in the event of an attack^4^. Because the ability of plants to detect and respond to VOCs that are emitted by neighbors following herbivore attack was discovered in alders, willows, and poplars^5,6^, it was initially dubbed the “talking trees” phenomenon^7^. However, because the emitting plant does not benefit from the receiver's response, the detection of emitted volatiles is more akin to receivers “eavesdropping” than emitters “talking”^8^. The term “signal” is used to distinguish infochemicals that are generated to send a message to a receiver, and the term “cue” is used when there is no intention to send a message^3^.

Like terrestrial plants, marine macroalgae (seaweeds) produce a variety of VOCs, including sulfonium compounds and halogenated and non-halogenated terpenes, aldehydes, acids, alcohols, amines, amides, and other hydrocarbons^9,10^. Their release is affected by abiotic factors such as irradiance, exposure to ultraviolet light, desiccation, and changes in seawater pH, salinity, and nutrient concentrations^11^. Seaweed volatiles can also be released into the air or water during reproduction and when herbivores consume seaweeds^12^. Many of these compounds have secondary functions. For example, macroalgal VOCs can deter feeding by herbivores^13,14^ and inhibit microbial growth^15^.

Inter-individual communication in marine seaweeds is similar to that in terrestrial plants, except that the transport of infochemicals typically occurs in water rather than air. In marine macroalgae, the detection of waterborne cues generated during grazing on seaweeds by neighboring conspecifics was first described in the brown alga *Ascophyllum nodosum*^16^. After 20 days, *A. nodosum* downstream of conspecifics being grazed by snails (*Littorina obtusata*) had higher phlorotannin concentrations. They were also less palatable to *L. obtusata* than *A. nodosum* downstream of ungrazed conspecifics. Crab (*Carcinus maenas*) and fish (*Lipophrys pholis*) predators of *L. obtusata* were later found to preferentially orient towards previously grazed *A. nodosum*, even when the grazers were absent, indicating predators also detect chemical cues emitted by grazed seaweeds^17^. Subsequently, waterborne cues released during grazing have been shown to reduce palatability in conspecific neighbors of other seaweed species, including the brown algae *Fucus vesiculosus*^18^, the red algae *Polyoides rotundus*^19^ and *Laurenica dendroidea*^20^, and the green algae *Cladophora rupestris* and *Ulva lactuca*^18^, with response times ranging from 72 h^20^ to 14 days^19^.

Even though intertidal seaweeds are marine organisms, they spend part of their lives emersed during low tides, a time when they can be functionally similar to terrestrial plants. During emersion, seaweeds are subject to biotic and abiotic stressors^21^ that can cause the release of VOCs into the air^11^. Thus, neighbors may be able to detect these compounds, use them as indicators of the emitter's condition or environment, and respond to them by increasing the production of antioxidant or antiherbivore defenses. Support for the ability of seaweeds to detect and respond to airborne cues in an ecologically relevant manner is much more limited than it is for waterborne cues. Increases in phlorotannin concentrations to levels comparable to those induced by artificial damage have been shown to occur in the brown alga *F. vesiculosus* 10 to 14 days after brief exposures to 5.4 to 540 nM methyl jasmonate^22^. Methyl jasmonate and related oxylipins, including jasmonic acid, are infochemicals used by terrestrial plants^23^. However, methyl jasmonate is not known to occur in *F. vesiculosus*^22^, and a subsequent study found no evidence of jasmonic acid in *F. vesiculosus* or six other seaweeds^14^. Thus, while *F. vesiculosus* has the physiological capacity to detect and respond to methyl jasmonate, the response might not be ecologically relevant.

Here, we describe laboratory and field experiments that we conducted to determine whether the intertidal green alga *Ulva fenestrata* can detect and respond to airborne dimethyl sulfide (DMS) and acrylic acid, VOCs that are breakdown products of dimethylsulfoniopropionate (DMSP)^24^. DMS and acrylic acid act as antiherbivore and antioxidant defenses in this alga and are released when it is exposed to biotic and abiotic stressors^25,26^.

## Methods

### Study organisms and chemistry

*U. fenestrata* (previously *U. lactuca*) is a green alga (Phylum Chlorophyta; Order Ulvales) with a distromatic membranous blade that grows abundantly in the mid to low intertidal in Washington’s inland marine waters^27,28^. It is diplobiontic with isomorphic sporophytes and gametophytes. Like many green macroalgae, *U. fenestrata* produces high concentrations of DMSP^29^, a sulfonium compound found in many marine microalgae, macroalgae, and invertebrates^30^. In both macroalgae and microalgae, DMSP is cleaved by an enzyme (or enzymes) into DMS and acrylate or acrylic acid^29,31^. Typical DMSP concentrations in northeastern Pacific *U. fenestrata* are 300–500 µmol g^−1^ of the alga's dry mass; however, there can be extensive spatial and temporal differences in concentrations^26,32^.

Ulvoid algae are among the least preferred foods of the sea urchins *Strongylocentrotus droebachiensis* and *Strongylocentrotus purpuratus*^33^. In northeastern Pacific macroalgae, one of the primary functions of DMSP is grazer deterrence via an activated antiherbivore defense^13,29^. Grazing and other physical damage cause DMSP to be rapidly cleaved into acrylic acid and DMS^13,34^, which deter feeding by *S. droebachiensis*^13,29,35^. A second and probably equally important function of DMSP in ulvoid algae is rapidly generating DMS and acrylic acid to alleviate oxidative stress^36^. Stressors such as hyposaline conditions and desiccation cause ulvoid algae to cleave DMSP into DMS and acrylic acid, which are both stronger antioxidants than DMSP^36,37^. Hydrogen peroxide, a chemical oxidant, can induce higher DMSP concentrations in lower intertidal ulvoid species, including *U. fenestrata*, which are subjected to less frequent physiological stresses than higher intertidal species^38^. In higher intertidal ulvoid species, changes in DMSP concentrations do not occur in response to increased concentrations of hydrogen peroxide in the surrounding seawater.

### Laboratory experiment

**Figure 1:**
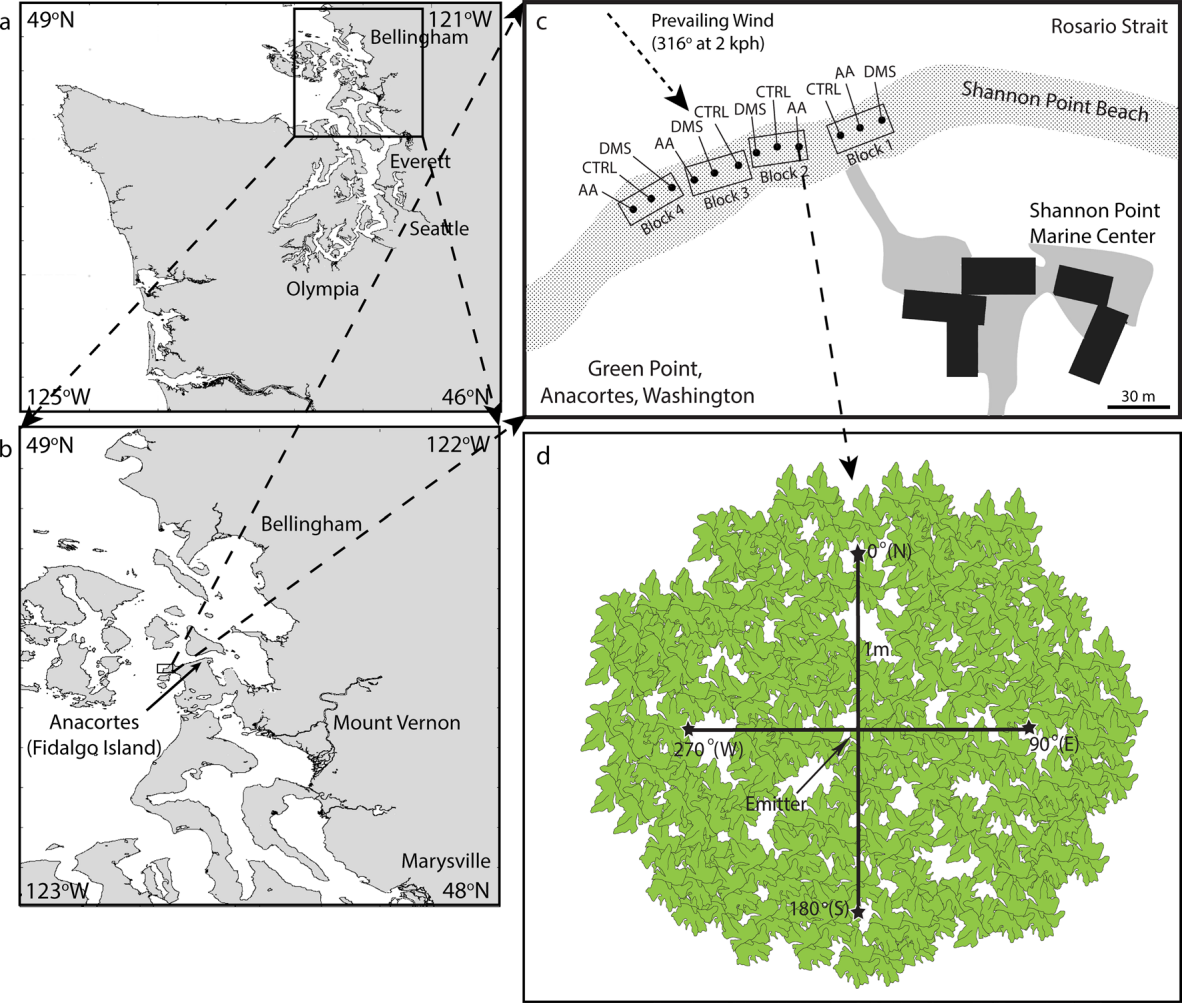
Location and design of the field experiment on the Shannon Point Beach, Anacortes, Washington, USA (**c**). The site was located in the town of Anacortes on Fidalgo Island (**b**) in Washington State (**a**). The map in c shows the four blocks, each with dimethyl sulfide (DMS), acrylic acid (AA), and control (CTRL) emitters on the Shannon Point Beach. Emitters were placed in the centers of *U. fenestrata* patches (**d**) and left for three h during a low tide. Black stars in panel d indicate areas where *U. fenestrata* were sampled 1 m north (N), east (E), south (S), and west (W) of the location of the emitter 1, 3, 7 and 11 days later. The coast outlines for panels a and b were obtained with the GEODAS Coastline Extractor (https://www.ncei.noaa.gov/products/marine-trackline-geophysical-data).

To examine the effects of DMS in a controlled setting, 500 circular disks of approximately 1 cm diam (5 mg fresh mass per disk) were punched with a cork borer from fifty *U. fenestrata* collected from the cobble beach in front of the Shannon Point Marine Center (Fig. 1; 48º 31’ N, 122º 41’ W), Anacortes, WA, USA (hereafter referred to as the Shannon Point Beach). The disks were placed in several bowls, each containing one L of f/2 culture medium^39^. The bowls were put in a lighted incubator (12 °C, 12:12 light: dark, 43 µmol photons^.^m^−2^ s^−1^) for 8 days to allow the algae to recover from being cut and to acclimate them to the culture conditions. During the acclimation period, the culture medium was changed every 3 days.

After acclimation, 240 disks were haphazardly selected from the bowls and placed on the bottoms of four 20 L black buckets (N = 60 per bucket). Black buckets were used to prevent DMS from breaking down due to light exposure. An additional 24 disks were haphazardly selected for pre-exposure DMSP measurements. The disks in the buckets were lightly misted with seawater, and a 4 cm bowl with 10 ml of seawater was added to each bucket to keep the humidity high and prevent the disks from desiccating. In two of the four buckets, 15 µl of DMS was added to create a ten µmol/L concentration. Two control buckets had no DMS added. Assuming that all the DMS in the buckets evaporated, the DMS concentrations in the treatment buckets were comparable to concentrations measured when sea urchins grazed on ulvoid algae in laboratory experiments^13^ and about 20% of concentrations when snails (*Littorina sitkana*) grazed on ulvoid macroalgae^34^. Immediately after the DMS was added, the buckets were sealed with Gamma Seal™ lids to make them airtight and prevent DMS from escaping, and moved to a 15 °C cold room.

After six h of exposure, the algae from each bucket were removed and placed in two 20 cm diam glass bowls containing one L of f/2 culture medium (N = 30 algal pieces per bowl; 8 bowls total). Algae were removed from control buckets first to avoid exposure to DMS from the experimental treatments. Three algal disks were haphazardly removed from each bowl for day 0 DMSP measurements. The bowls were then placed in a lighted incubator (12 °C, 12:12 light: dark, 43 µmol photons m^−2^ s^−1^) for 15 days. One, three, six, nine, twelve, and fifteen days following DMS exposure, three disks were haphazardly selected from each bowl for DMSP measurements.

To measure DMSP, algal pieces (5–10 mg) were dried in an oven overnight at 60 °C. Measuring DMSP in dried green algae has been shown to generate higher DMSP concentrations than conducting analyses on fresh material^40^. After they dried, the algal pieces were weighed and placed in 4 mL of 4 N sodium hydroxide (NaOH) in 30 ml gas-tight vials. The following day, headspace DMS was measured on an SRI gas chromatograph (10 µl injection, Chromosil 330 column at 90 °C, FPD). Commercially obtained DMSP (Center for Analysis, Spectroscopy and Synthesis, University of Groningen, purity > 0.98) was used as a standard. All DMSP concentrations were normalized to the sample’s dry mass, and the analysis's detection limit was 12.5 µg DMSP per 30 mL vial.

### Field experiment

An initial field experiment was conducted in which DMS and acrylic acid were released in the intertidal zone, and DMSP concentrations in neighboring algae were monitored for 11 days. These experiments were conducted on the Shannon Point Beach. This cobble beach is moderately protected from large swells and waves by its proximity to Cypress and Guemes Islands to the north and northeast, the San Juan Islands to the west, and Fidalgo Head to the south and southeast. *U. fenestrata* is abundant on the beach from late spring through mid-fall but is rarely found during the winter.

DMS, acrylic acid, and control emitters were placed in 12 patches of *U. fenestrata* located at mean lower low water (MLLW) in the intertidal zone on the morning of 16 Jul 2007. The patches were aligned horizontally along the beach so that they were at the same tidal level and were separated from one another by at least 10 m (Fig. 1c). They were grouped into four experimental blocks, each having a control emitter, a DMS emitter, and an acrylic acid emitter, which were randomly ordered within each block. The emitters consisted of 60 ml Falcon™ tubes into which six holes were drilled to allow volatile compounds to escape. DMS and acrylic acid were added to the emitters by pipetting 1 ml of each onto a cotton ball and putting it into the bottom of the tube. In the control emitters, no compounds were added. The tubes were capped and placed in an inverted position (balanced on the cap) on flat rocks in the intertidal zone. An additional 1 ml of DMS and acrylic acid was added into each emitter one and two hours after the emitters were deployed. The emitters were removed 3 h after deployment before the tide came in. The amounts of DMS and acrylic acid emitted were not measured but were likely much higher than would be released during herbivore grazing. However, this experiment aimed to determine whether *U. fenestrata* responds to the compounds in the field rather than whether it responds to natural concentrations. During the 3-h exposure, winds were light (mean wind speed: 2.1 km/h) and from the northwest (Fig. 1c), blowing at an angle of approximately 80° to the beach, which is aligned in an ESE to WSW direction.

Two *U. fenestrata* were sampled 1 m north, south, east, and west around the spots where the emitters were deployed on the day of deployment (Fig. 1d), and during low tides one, two, three, seven, and eleven days thereafter. A 50–100 mg piece of each sampled alga was weighed and dried in a 60 °C oven overnight for later analysis of tissue DMSP concentrations, which were measured as described for the laboratory experiment.

### Statistical analyses

For the laboratory experiment, pre-treatment and immediately post-treatment DMSP concentrations were compared with ANOVA (Minitab 2022, General Linear Model) after ensuring the data met the assumption of normality (Anderson–Darling test: P = 0.193). Factors in the analysis included sampling time (pre- or post-treatment), treatment (DMS versus control), and bowls (nested within buckets). The DMSP concentrations from days 1 to 15 of the laboratory experiment did not meet the assumption of normality (Anderson–Darling test); therefore, the data were Box-Cox transformed using an optimal λ of 0.431. An analysis of variance (Minitab 2022 General Linear Model) was then used to determine whether there were significant differences (α = 0.05) among DMSP concentrations based on sampling date, treatments (DMS added versus control), and bowls (nested within buckets). To investigate the effects of adding DMS on DMSP concentrations for each sampling day, algal pieces in the control buckets were randomly paired with algal pieces in the buckets to which DMS was added using the RAND function in Excel to generate pseudo-random numbers. The percent change in DMSP concentrations in the DMS-exposed pieces relative to the DMSP in the control pieces was then calculated. Percent increases in DMSP were compared to an expected value of 0 with a one-tailed t-test if the data were normally distributed and with a Wilcoxon test if they were not. An Anderson–Darling test was used to determine whether the data met the assumption of normality.

Because DMSP concentrations from the field experiment did not meet the assumption of normality (Anderson–Darling test), the data were Box-Cox transformed using an optimal λ of 0.624 prior to analyses. An analysis of variance (ANOVA; Minitab 2022, General Linear Model) was used to determine whether there were significant differences (α = 0.05) among DMSP concentrations based on sampling date, experimental blocks, emitter types (control, acrylic acid, or DMS, nested within experimental blocks) and whether the samples were obtained upwind (north or west) or downwind (east or south) of the emitter. DMSP concentrations were then examined separately for each collection date with ANOVA to determine the effects of block, emitter type, and sampling direction. Finally, DMSP concentrations of algae collected on Day 11 were examined to determine the effects of blocks and sampling direction for each emitter type separately.

## Results

### Laboratory experiment

**Figure 2:**
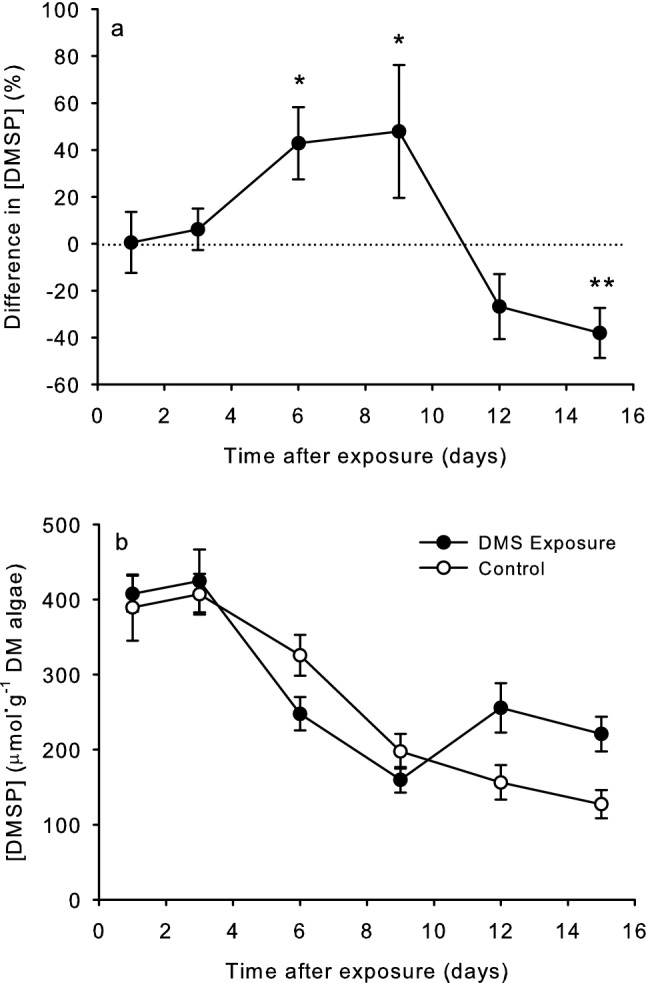
Effect of exposure to dimethyl sulfide (DMS) in laboratory experiments over time. (**a**) Data as means (± 1 SE) of percent increases in randomly paired pieces of *U. fenestrata* exposed to DMS and control pieces not exposed to DMS. * P < 0.05 from t-tests (normally distributed data) or Wilcoxon’s test (non-normally distributed data) ** P < 0.01. (**b**) Data as means (± 1 SE) of DMSP concentrations in *U. fenestrata* exposed to DMS and control pieces not exposed to DMS.

In the laboratory experiments, *U. fenestrata* exposed to DMS initially had similar DMSP concentrations as controls (Fig. 2a,b, Table 1; day one t-test: T = 0.04, P = 0.966; day three t-test: T = 0.69, P = 0.504). Although DMSP concentrations generally declined during the first 9 days (Fig. 2b), during days six and nine, DMSP concentrations in DMS-exposed seaweeds were 43–48% higher than in controls (Fig. 2a; day six t-test: T = 2.79, P = 0.018; day nine Wilcoxon test: Wilcoxon statistic = 69, P = 0.021). This pattern reversed on days 12 and 15 when DMS-exposed *U. fenestrata* had DMSP concentrations that averaged 27–38 percent lower than controls (Fig. 2a; day 12 Wilcoxon test: Wilcoxon statistic = 17, P = 0.092; day 15 t-test: T = − 3.59, P = 0.004). There were also no significant differences in DMSP concentrations in *U. fenestrata* before and immediately after the algae were exposed to DMS on the day the exposures took place (Table 2; ANOVA treatment effect: df = 1, F = 3.02, P = 0.09).

**Table 1:** Analysis of variance to examine the effects of sampling days (1, 3, 6, 12, and 15), treatment (control and DMS), and bowls (nested within buckets) on Box-Cox transformed (λ = 0.431) DMSP concentrations of *U. fenestrata* in the laboratory experiments.

Source	df	SS	MS	F	P
Day	5	14.8369	2.96738	25.94	** < 0.001**
Treatment	1	0.2358	0.23581	2.06	0.153
Bowl (Bucket)	4	0.3196	0.07990	0.70	0.594
Day x treatment	5	2.5331	0.50662	4.43	**0.001**
Error	128	14.6405	0.11438		
Total	143	32.5659			

Significant values are in bold.

**Table 2:** Analysis of variance to examine the effects of sampling time (before and immediately after exposure on the day of the exposure experiment), treatment (control and DMS), and bowls (nested within buckets) on DMSP concentrations of *U. fenestrata* in the laboratory experiments.

Source	df	SS	MS	F	P
Time	1	0.342	0.3417	0.07	0.791
Treatment	1	14.553	14.5530	3.02	0.090
Time x treatment	1	0.955	0.9549	0.20	0.659
Bowl (Bucket)	4	40.791	10.1979	2.12	0.097
Error	40	192.783	4.8196		
Total	47	249.424			

### Field experiment

**Figure 3:**
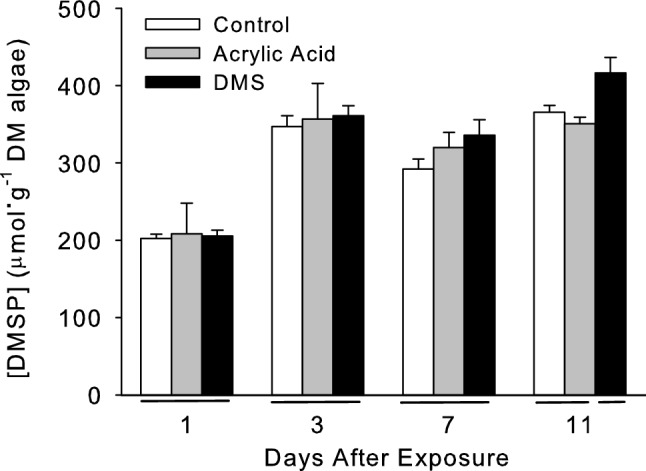
Mean concentration (± 1 SE) of DMSP (as µmol DMSP per g of algal dry mass [DM]) in *U. fenestrata* after exposure to acrylic acid (grey bars), and DMS (black bars) 1, 3, 7 and 11 days after exposure to the compounds. White bars are DMSP concentrations near control emitters. Horizontal bars below the x-axis indicate means that are not significantly different from one another (post hoc Tukey’s test, P > 0.05).

**Figure 4:**
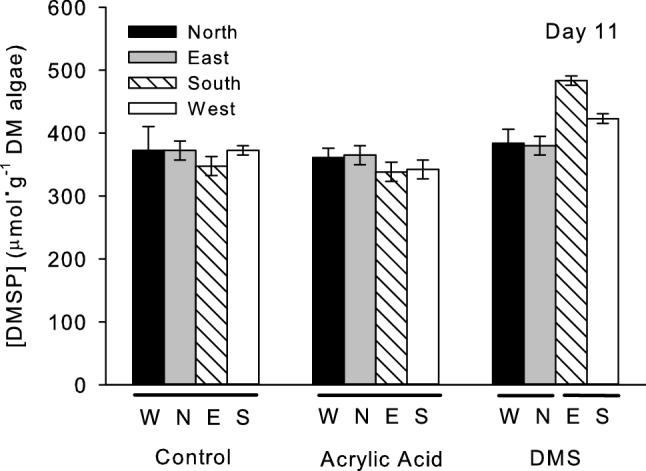
Mean concentration (± 1 SE) of DMSP (as µmol DMSP per g of algal dry mass [DM]) in *U. fenestrata* eleven days after exposure to airborne acrylic acid and dimethyl sulfide (DMS). Algae were collected 1 m to the west (W; black bars), north (N; grey bars), east (E; hatched bars), and south (S; white bars) of the emitters' locations. Horizontal bars below the x-axis indicate means that are not significantly different from one another (post hoc Tukey's test, P > 0.05).

In the field experiment, DMSP concentrations in *U. fenestrata* were significantly higher in algae near the DMS emitters (Fig. 3, Table 3, post hoc Tukey’s test: P < 0.05) than in *U. fenestrata* near the control and acrylic acid emitters, which did not differ from one another (post hoc Tukey’s test, P > 0.05). DMSP concentrations also differed significantly among blocks, days, and emitter types (Table 3). This was largely a result of higher DMSP concentrations in algae downwind of the DMS emitters on day 11 (Fig. 4), particularly east of the DMS emitter, where average DMSP concentrations were 35% higher than the average near the control and acrylic acid emitters. When the data for each day were examined separately, concentrations differed significantly among the different types of emitters on day 11 (Table S1; ANOVA emitter type effect: df = 2, F = 3.33, P = 0.003) but not on days one (Table S2; ANOVA emitter type effect: df = 2, F = 0.97, P = 0.465), three (Table S3; ANOVA emitter type effect: df = 2, F = 1.53, P = 0.163), and seven (Table S4; ANOVA emitter type effect: df = 2, F = 1.33, P = 0.243). DMSP concentrations in *U. fenestrata* downwind of the DMS emitters (east and south) were, on average, 19% higher than those upwind (north and west) of the emitters (Table S5; ANOVA location effect: df = 1, F = 6.10, P = 0.024).

**Table 3:** Analysis of variance to examine the effects of blocks, sampling days (1, 3, 7 and 11), emitter types (control, acrylic acid, DMS) nested within blocks, and sampling direction relative to the emitters (upwind, downwind) on Box-Cox transformed (λ = 0.624) DMSP concentrations of *U. fenestrata* in the field experiments.

Source	df	SS	MS	F	P
Block	3	8.793	2.9310	16.75	** < 0.001**
Day	4	75.539	18.8848	107.92	** < 0.001**
Direction	1	0.288	0.2880	1.65	0.200
Block*day	12	42.362	3.5301	20.17	** < 0.001**
Emitter type (Block)	8	7.919	0.9899	5.66	** < 0.001**
Block x direction	3	0.197	0.0656	0.38	0.771
Day x direction	4	0.535	0.1337	0.76	0.549
Day x emitter type (Block)	32	13.545	0.4233	2.42	** < 0.001**
Block x day x direction	12	3.745	0.3121	1.78	0.049
Emitter type (Block) x direction	8	1.368	0.1711	0.98	0.453
Day x emitter type (Block) x direction	32	10.832	0.3385	1.93	**0.002**
Error	360	62.998	0.1750		
Total	479	228.122			

Significant values are in bold.

## Discussion

### DMS as an infochemical

DMSP is a multifunctional molecule^24^ found in many photosynthetic organisms, from phytoplankton to seaweeds to vascular plants^41^. It is also produced by symbiotic microalgae harbored by many marine invertebrates, such as stony corals, soft corals, and tridacnid clams^42^. Because of its diverse ecological roles in oceanic ecosystems, including its ability to function as an infochemical, and its importance to the global sulfur cycle^43^, DMSP has been designated a “molecule of keystone significance”^44^.

While the functions of DMSP and its products in the organisms that produce it are not always well understood, it is clear that many environmental factors affect their production^45,46^. Our study has elucidated a novel role for DMS, namely as an airborne infochemical in marine interplant communication, and adds to the known infochemical functions of DMS and DMSP. Many animals use the smell of DMS and DMSP for foraging and navigating. DMS released by grazed phytoplankton is used by meso- and microzooplankton as a foraging cue^47^. Oceanic procellariform seabirds use DMS released by grazed microalgae to find concentrated patches of prey^48^, and juvenile loggerhead turtles can detect DMS, which may help them orient towards favorable foraging areas^49^. Tropical planktivorous reef fishes orient towards plumes of DMSP, allowing them to find productive reef habitats^50^, and larval reef fish orient towards DMS and exhibit exploratory swimming behaviors, which may help them find food and favorable areas for settlement^51^.

The microbial community associated with *Ulva* may also play a role in DMS-mediated communication. Seaweeds harbor a diverse community of microorganisms^52^, including bacterial species that produce low molecular weight metabolites critical to their hosts’ morphological development^53^. In laboratory assays, DMSP attracts cells of the bacterium *Roseovarius* sp. MS2^54^, a strain known to produce morphogenetic compounds that cause *Ulva mutabilis* to form blades rather than calluses^53^. *Roseovarius* and other bacteria that are known to associate with marine benthic organisms can catabolize DMSP and generate DMS^54,55^. Other marine bacteria, including those known to be associated with sediments, produce DMSP^56^. Whether microbes associated with *U. fenestrata* are involved in producing, detecting, and responding to airborne DMS is not known but could be an interesting area for future studies.

The timing of the DMSP increases in our experiments was comparable to the three^20^ to fourteen^19^ day range reported for other types of induced defenses in seaweeds triggered by waterborne cues. However, the increases we observed occurred later in our field than in our laboratory experiments. Proportional increases were also larger in magnitude in our laboratory experiments. The differences in timing in our laboratory and field experiments could be due to differences in the environments that the seaweeds experienced. Thomas et al.^57^ found the induction of defense characteristics in response to oligoguluronate elicitors in the kelp *Laminaria digitata* differed in laboratory-reared versus wild kelp. When laboratory-reared *L. digitata* were transplanted into the wild, responses to elicitors became similar to wild kelp, suggesting that waterborne environmental cues primed the algae for a faster response to elicitors. The more rapid response we saw in the laboratory could have been a result of being exposed to more concentrated elicitors in the buckets relative to the field, the more benign environment in the laboratory, or other environmental cues present in the field that altered the alga’s response.

DMSP concentrations in ulvoid algae are also dynamic—temporal changes in concentrations are affected both by the synthesis of DMSP as well as by its catabolism, which can occur in response to damage from grazing^13^, environmental stress^15^ and possibly other yet to be described environmental cues. Thus, the increases in DMSP concentrations we observed relative to controls could result from increased production of DMSP or reductions in its catabolism. Furthermore, environmental cues may affect rates of production and catabolism differently. If environmental cues affect both DMSP production and catabolism, but the timing of those responses differs, then DMSP concentrations in affected algae will change over time. This could explain the initial increase in DMSP concentrations followed by a later reduction on days 12–15 in our laboratory experiments. Alternatively, the drop in DMSP concentrations on days 12–15 could result from reductions in DMSP production driven by an exhaustion of resources used to produce the spike in concentrations on days 6–9. The ability to respond to waterborne or airborne cues may also be affected by upper limits to the concentrations of DMSP that can be maintained within an organism. The highest comparable reported value for DMSP concentrations in *U. fenestrata* in the region where we conducted our experiments is 500 µmol g^-1^ DM of algae^38^, only slightly above the maximum average concentration we measured in algae downwind of DMS emitters in our field experiment. Therefore, it is possible that the algae in our field experiment were already close to their maximum concentrations and could not exceed these amounts.

### Eavesdropping in seaweeds

Our results also demonstrate that *U. fenestrata* can “eavesdrop”, i.e. detect an airborne VOC released by conspecifics when injured and respond by increasing concentrations of a defensive metabolite. To our knowledge, this is the first demonstration that marine macroalgae or marine plants use airborne infochemicals known to be released during grazing to elicit responses that may be defensive. As the green seaweeds and land plants share a common ancestor^58^, our results suggest that the ability to detect airborne compounds either arose prior to the evolution of terrestrial plants or evolved multiple times.

As for many land plants, this function is likely not occurring for the benefit of the emitter when the emitter and receiver are unrelated individuals. Rather, the emitter benefits from the breakdown of DMSP and release of DMS and acrylic acid into the environment because these breakdown products deter feeding by herbivores^13,29^ and function as antioxidants, scavenging reactive oxygen species (ROS) released by the alga when physically damaged or physiologically stressed^36^. However, detecting DMS and using it as a signal to increase DMSP concentrations would confer an evolutionary advantage to the receivers if increased DMSP concentrations provide the receiver with better protection against herbivores or improve their ability to scavenge ROS following damage or oxidative stress.

However, if the emitter and receiver are related, either because the receiver is a tissue in another part of the thallus or is an independent fragment (i.e. clone) of the emitter, then the emitter would benefit from releasing chemical signals that increase defenses in receivers. Some land plants, especially those with poorly developed vascular systems^59^, use airborne signals to communicate amongst leaves^60^. In general, seaweeds have poorly developed or no vascular systems^61^, and like many *Ulva* spp., *U. fenestrata* fragments easily, with fragments of seaweeds continuing to grow and reproduce^62^. Therefore, emitting airborne DMS and other chemical signals may have evolved as intra-plant signaling mechanisms. In this scenario, an attack on one part of the thallus would result in the emission of waterborne or airborne signals, which initiate increases in defenses in distant areas of the thallus or clonal fragments. In this case, seaweeds could be considered to be “talking” as the emitter would gain a fitness benefit by releasing a signal that benefits another part of itself or genetically identical clones.

The ability to respond to signals or cues generated by physiological stress and to induce increased antioxidant capacity might be particularly important for ephemeral intertidal seaweeds that have limited growing seasons and live in habitats with pronounced environmental stress gradients. *U. fenestrata* and related ulvoid seaweeds in the Pacific Northwest tend to be uncommon in the winter, then recruit and grow rapidly in the spring^27^, with biomasses being highest in late summer and early autumn. Tidal ranges in the area are 3–4 m (https://eopugetsound.org/articles/puget-sound-tides), creating pronounced physiological stress gradients. Furthermore, ulvoid algae tend to be found in areas with limited water motion. They are often unattached^63^ and can be moved by tides and currents to distant locations where they encounter changes in herbivore density or differences in physiological stresses. Having the ability to eavesdrop on infochemicals generated by other seaweeds would allow them to rapidly upregulate defenses as conditions change seasonally, when they drift into areas with more stressful conditions, or following episodic recruitments of herbivores.

While intraspecific communication, in which the emitter and receiver are members of the same species, is the norm in terrestrial plants, examples of interspecific communication exist^64,65^. Four species of ulvoid algae, *Ulva intestinalis*, *Ulvaria obscura*, *U. fenestrata*, and *Ulva linza*, can co-occur on northeastern Pacific beaches, and all produce DMSP and DMS^25,26^. Although these four species tend to grow in different zones on the shore, there is overlap in their vertical distributions, with *U. fenestrata* zoned between *U. linza* and *U. obscura*, and *U. intestinalis* typically found highest on the shore, often near freshwater seeps^27,66,67^. *U. intestinalis*, *U. obscura*, and *U. fenestrata* have been shown to release DMS in response to hyposaline conditions, high temperatures, and desiccation. However, the amounts emitted differ among species and stressors, with the highest emissions produced by desiccated *U. intestinalis*^25^. Consequently, DMS may act as an interspecific cue that can be emitted and detected by multiple green seaweed species living near one another. Furthermore, *U. fenestrata* and *U. obscura* have been shown to release ROS into the surrounding seawater when physiologically stressed, especially following desiccation during emersion^68^, and DMSP concentrations in *U. fenestrata* and *U. obscura*, but not in *U. linza* and *U. intestinalis*, are induced in response to waterborne ROS^38^. Hence, *U. fenestrata* can increase DMSP concentrations in response to both airborne DMS and waterborne ROS, which could originate not only from conspecifics but also from other ulvoid species.

Our study has demonstrated that an intertidal seaweed responds to a VOC that it and many other intertidal seaweeds produce by increasing concentrations of a defensive metabolite. Given the wide variety of VOCs released by intertidal macroalgae^9–12^, including plant hormones^69,70^, the ability to respond to them may be a common mechanism for transmitting information and regulating physiological processes between individuals and tissues, within and among species.

## Supplementary Information

Supplementary Tables.

## Data Availability

The datasets generated for this study are available in the figshare repository at https://doi.org/10.6084/m9.figshare.21350238.v1

## References

[1] Heil M, Karban R (2010). Explaining evolution of plant communication by airborne signals. Trends Ecol. Evol..

[2] Karban R (2021). Plant communication. Ann. Rev. Ecol. Evol. Syst..

[3] Ninkovic V, Markovic D, Rensing M (2021). Plant volatiles as cues and signals in plant communication. Plant Cell Environ..

[4] Zhou S, Jander G (2022). Molecular ecology of plant volatiles in interactions with insect herbivores. J. Exp. Bot..

[5] Baldwin IT, Schultz JC (1983). Rapid changes in tree leaf chemistry induced by damage: Evidence for communication between plants. Science.

[6] Rhoades DF, Hedin PA (1983). Responses of alder and willow to attack by tent caterpillars and webworms: evidence for pheromonal sensitivity of willows. Plant Resistance to Insects.

[7] Fowler SV, Lawton JH (1985). Rapidly induced defenses and talking trees: The devil's advocate position. Amer. Nat..

[8] Baldwin IT, Halitschke R, Paschold A, Von Dahl CC, Preston CA (2006). Volatile signaling in plant-plant interactions: "Talking trees" in the genomics era. Science.

[9] Neta MTSL, Narain N (2018). Volatile components in seaweeds. Examines Mar. Biol..

[10] Pozzer A, Gómez P, Weiss J (2022). Volatile organic compounds in aquatic ecosystems—Detection, origin, significance and applications. Sci. Total Environ..

[11] Keng FSL (2020). The emission of volatile halocarbons by seaweeds and their response towards environmental changes. J. Appl. Phycol..

[12] Saha M, Fink P (2022). Algal volatiles–the overlooked chemical language of aquatic primary producers. Biol. Rev..

[13] Van Alstyne KL, Houser LT (2003). Dimethylsulfide release during macroinvertebrate grazing and its role as an activated chemical defense. Mar. Ecol. Prog. Ser..

[14] Weisemeier T, Hay M, Pohnert G (2007). The potential role of wound-activated volatile release in the chemical defence of the brown alga *Dictyota dichotoma*: blend recognition by marine herbivores. Aquat. Sci..

[15] Ozdemir G, Horzum Z, Sukatar A, Karabay-Yavasoglu NU (2006). Antimicrobial activities of volatile components and various extracts of *Dictyopteris membranaceae* and *Cystoseira barbata*. From the coast of Izmir, Turkey. Pharm. Biol..

[16] Toth G, Pavia H (2000). Water-borne cues induce chemical defense in a marine alga (*Ascophyllum nodosum*). Proc. Nat. Acad. Sci..

[17] Coleman R, Ramchunder S, Davies K, Moody A, Foggo A (2007). Herbivore-induced infochemicals influence foraging behaviour in two intertidal predators. Oecologia.

[18] Yun H, Cruz J, Treitschke M, Wahl M, Molis M (2007). Testing for the induction of anti-herbivory defences in four Portuguese macroalgae by direct and waterborne cues of grazing amphipods. Helgol. Mar. Res..

[19] Toth G (2007). Screening for induced herbivore resistance in Swedish intertidal seaweeds. Mar. Biol..

[20] Pereira R (2020). The sea-hare *Aplysia brasiliana* promotes induction in chemical defense in the seaweed *Laurencia dendroidea* and in their congeneric neighbors. Plant Physiol. Biochem..

[21] Hurd CL, Harrison PJ, Bischof K, Lobban CS (2014). Seaweed Ecology and Physiology.

[22] Arnold TM, Targett NM, Tanner CE, Hatch WI, Ferrari K (2001). Evidence for methyl jasmonate-induced phlorotannin production in *Fucus vesiculosus* (Phaeophyceae). J. Phycol..

[23] Ruan J (2019). Jasmonic acid signaling pathway in plants. Internat J. Mol. Sci..

[24] Stefels J (2000). Physiological aspects of the production and conversion of DMSP in marine algae and higher plants. J. Sea Res..

[25] Van Alstyne KL, Gifford S-A, Dohman J, Savedo M (2015). Effects of environmental changes, tissue types, and reproduction on emissions of dimethyl sulfide from seaweeds that form green tides. Environ. Chem..

[26] Van Alstyne KL, Nelson TA, Ridgway RL (2015). Environmental chemistry and chemical ecology of "green tide" seaweed blooms. Int. Comp. Biol..

[27] Nelson TA, Nelson AV, Tjoelker M (2003). Seasonal and spatial patterns of "green tides" (ulvoid algal blooms) and related water quality parameters in the coastal waters of Washington State, USA. Bot. Mar..

[28] Nelson, T. A., Olson, J. & Imhof, L. Using underwater video analysis to determine ulvoid cover and overlap with eelgrass over a regional scale. *Proceedings of the 2009 Puget Sound Georgia Basin Ecosystem Conference* 8–11 (2009).

[29] Van Alstyne KL, Wolfe GV, Freidenburg TL, Neill A, Hicken C (2001). Activated defense systems in marine macroalgae: Evidence for an ecological role for DMSP cleavage. Mar. Ecol. Prog. Ser..

[30] Van Alstyne KL, Amsler C (2008). Ecological and physiological roles of dimethylsulfoniopropionate (DMSP) and its DMSP cleavage in marine macroalgae. Algal Chemical Ecology.

[31] Alcolombri U (2015). Identification of the algal dimethyl sulfide–releasing enzyme: A missing link in the marine sulfur cycle. Science.

[32] Van Alstyne KL, Koellermeier L, Nelson T (2007). Spatial variation in dimethylsulfoniopropionate (DMSP) production in *Ulva lactuca* (Chlorophyta) from the Northeast Pacific. Mar. Biol..

[33] Van Alstyne KL, Nelson AV, Vyvyan JR, Cancilla D (2006). Dopamine functions as an antiherbivore defense in the temperate green alga *Ulvaria obscura*. Oecologia.

[34] Van Alstyne KL, Pelletreau KN, Kirby A (2009). Nutritional preferences override chemical defenses in determining food choice by a generalist herbivore, *Littorina sitkana*. J. Exp. Mar. Biol. Ecol..

[35] Lyons DA, Van Alstyne KL, Scheibling RE (2007). Anti-grazing activity and seasonal variation of dimethylsulfoniopropionate-associated compounds in the invasive alga Codium fragile ssp. tomentosoides. Mar. Biol..

[36] Ross C, Van Alstyne KL (2007). Intraspecific variation in stress-induced hydrogen peroxide scavenging by the ulvoid macroalga *Ulva lactuca*. J. Phycol..

[37] Sunda WK, Kieber DJ, Kiene RP, Huntsman S (2002). An antioxidant function for DMSP and DMS in marine algae. Nature.

[38] Van Alstyne KL, Sutton L, Gifford S-A (2020). Inducible versus constitutive antioxidant defenses along an environmental stress gradient. Mar. Ecol. Prog. Ser..

[39] Karsten U, Kuck K, Daniel C, Wiencke C, Kirst GO (1994). A method for complete determination of dimethylsulfoniopropionate (DMSP) in marine macroalgae for different geographical regions. Phycologia.

[40] Andersen RA (2005). Algal Culturing Techniques.

[41] Otte ML, Wilson G, Morris JT, Moran BM (2004). Dimethylsulphoniopropionate (DMSP) and related compounds in higher plants. J. Exp. Bot..

[42] Deschaseaux E, Jones G, Swan H (2015). Dimethylated sulfur compounds in coral-reef ecosystems. Environ. Chem..

[43] Jackson R, Gabric A (2022). Climate change impacts on the marine cycling of biogenic sulfur: A review. Microorganisms.

[44] Ferrer RP, Zimmer RK (2013). Molecules of keystone significance: Crucial agents in ecology and resource management. Bioscience.

[45] Caruana AM, Malin G (2014). The variability in DMSP content and DMSP lyase activity in marine dinoflagellates. Prog. Oceanogr..

[46] Burdett HL, Hatton AD, Kamenos NA (2015). Coralline algae as a globally significant pool of marine dimethylated sulfur. Global Biogeochem. Cycles.

[47] Shemi A (2021). Dimethyl sulfide mediates microbial predator–prey interactions between zooplankton and algae in the ocean. Nat. Microbiol..

[48] Nevitt GA (2011). The neuroecology of dimethyl sulfide: A global-climate regulator turned marine infochemical. Int. Comp. Biol..

[49] Endres CS, Lohmann KJ (2012). Perception of dimethyl sulfide (DMS) by loggerhead sea turtles: A possible mechanism for locating high-productivity oceanic regions for foraging. J. Exp. Biol..

[50] DeBose JL, Lema SC, Nevitt GA (2008). Dimethylsulfoniopropionate as a foraging cue for reef fishes. Science.

[51] Foretich MA, Paris CB, Grosell M, Stieglitz JD, Benetti DD (2017). Dimethyl sulfide is a chemical attractant for reef fish larvae. Sci. Rep..

[52] Egan S (2013). The seaweed holobiont: Understanding seaweed–bacteria interactions. FEMS Microbiol. Rev..

[53] Wichard T (2022). From model organism to application: Bacteria-induced growth and development of the green seaweed *Ulva* and the potential of microbe leveraging in algal aquaculture. Semin. Cell Develop. Biol..

[54] Kessler RW, Weiss A, Kuegler S, Hermes C, Wichard T (2018). Macroalgal–bacterial interactions: Role of dimethylsulfoniopropionate in microbial gardening by *Ulva* (Chlorophyta). Mol. Ecol..

[55] Tandon K (2020). Comparative genomics: dominant coral-bacterium *Endozoicomonas acroporae* metabolizes dimethylsulfoniopropionate (DMSP). ISME J..

[56] Williams BT (2019). Bacteria are important dimethylsulfoniopropionate producers in coastal sediments. Nature Microbiol..

[57] Thomas F (2011). Waterborne signaling primes the expression of elicitor-induced genes and buffers the oxidative responses in the brown alga *Laminaria digitata*. PLoS One.

[58] Lewis LA, McCourt RM (2004). Green algae and the origin of land plants. Amer. J. Bot..

[59] Frost CJ (2007). Within-plant signalling via volatiles overcomes vascular constraints on systemic signalling and primes responses against herbivores. Ecol. Lett..

[60] Gershenzon J (2007). Plant volatiles carry both public and private messages. Proc. Nat. Acad. Sci..

[61] Graham LE, Wilcox LW (2000). Algae.

[62] Bonneau ER (1978). Asexual reproductive capabilities in *Ulva lactuca* L. (Chlorophyceae). Bot. Mar..

[63] Merceron M, Morand P (2004). Existence of a deep subtidal stock of drifting *Ulva* in relation to intertidal algal mat developments. J. Sea Res..

[64] Farmer EE, Ryan CA (1990). Interplant communication: Airborne methyl jasmonate induces synthesis of proteinase inhibitors in plant leaves. Proc. Nat. Acad. Sci..

[65] Glinwood R, Ninkovic V, Pettersson J, Ahmed E (2004). Barley exposed to aerial allelopathy from thistles (Cirsium spp.) becomes less acceptable to aphids. Ecol. Entomol..

[66] Nelson TA (2000). Preliminary studies of seasonality, ecology, and species composition of ulvoid algal blooms in Washington State. J. Phycol..

[67] O'Clair RM, Lindstrom SC (2000). North Pacific Seaweeds.

[68] van Hees DH, Van Alstyne KL (2013). Effects of emersion, temperature, dopamine, and hypoxia on the accumulation of extracellular oxidants surrounding the bloom-forming seaweeds *Ulva lactuca* and *Ulvaria obscura*. J. Exp. Mar. Biol. Ecol..

[69] Plettner IN, Steinke M, Malin G (2005). Ethene (ethylene) production in the marine macroalga * Ulva * ( * Enteromorpha * ) intestinalis L. (Chlorophyta, Ulvophyceae): Effect of light-stress and co-production with dimethyl sulphide. Plant Cell Environ..

[70] Garcia-Jimenez P, Robaina RR, Kumar M, Ralph P (2017). Volatiles in the aquatic marine ecosystem: ethylene and related plant hormones and sporulation in red seaweeds. Systems Biology of Marine Ecosystems.

