# Supplementary material for: Airborne dimethyl sulfide (DMS) cues dimethylsulfoniopropionate (DMSP) increases in the intertidal green alga Ulva fenestrata
Source: Sci Rep. 2023 Mar 15;13:4298. doi: 10.1038/s41598-023-30881-9 (PMC10017803; doi:10.1038/s41598-023-30881-9)
Supplement: Supplementary file 1 — Supplementary Tables. [file 41598_2023_30881_MOESM1_ESM.pdf]

Supplemental Information

Table S1. ANOVA for Box-Cox transformed DMSP concentrations on day 11 of the field experiment. Treatments are DMS, acrylic acid, and solvent controls. Directions are north, east, west, and south of the emitters.

Source	df	SS	MS	F	P
Block	3	0.008148	0.002716	3.71	**0.015**
Direction	1	0.000088	0.000088	0.12	0.730
Treatment (Block)	8	0.019493	0.002437	3.33	**0.003**
Block x Direction	3	0.001397	0.000466	0.64	0.594
Treatment (Block) x Direction	8	0.016425	0.002053	2.80	**0.009**
Error	72	0.052728	0.000732		
Total	95	0.098279			

Table S2. Analysis of variance (ANOVA) for Box-Cox transformed DMSP concentrations on day 1 of the field experiment. Treatments are DMS, acrylic acid, and solvent controls. Directions are north, east, west, and south of the emitters.

Source	df	SS	MS	F	P
Block	3	59.118	19.706	4.77	**0.004**
Direction	1	1.321	1.321	0.32	0.573
Treatment (Block)	8	32.110	4.014	0.97	0.465
Block x Direction	3	76.147	25.382	6.14	**0.001**
Treatment (Block) x Direction	8	16.484	2.060	0.50	0.853
Error	72	297.484	4.132		
Total	95	482.663			

Table S3. ANOVA for DMSP concentrations on day 3 of the field experiment. Treatments are DMS, acrylic acid, and solvent controls. Directions are north, east, west, and south of the emitters.

Source	df	SS	MS	F	P
Block	3	3.548	1.1827	0.83	0.479
Direction	1	2.747	2.7473	1.94	0.168
Treatment (Block)	8	17.315	2.1643	1.53	0.163
Block x Direction	3	1.877	0.6257	0.44	0.724
Treatment (Block) x Direction	8	4.765	0.5956	0.42	0.905
Error	72	101.983	1.4164		
Total	95	132.234			

Table S4. ANOVA for Box-Cox transformed DMSP concentrations on day 7 of the field experiment. Treatments are DMS, acrylic acid, and solvent controls. Directions are north, east, west, and south of the emitters.

Source	df	SS	MS	F	P
Block	3	0.009739	0.003246	0.78	0.510
Direction	1	0.000314	0.000314	0.08	0.785
Treatment (Block)	8	0.044372	0.005546	1.33	0.243
Block x Direction	3	0.008430	0.002810	0.67	0.571
Treatment (Block) x Direction	8	0.033815	0.004227	1.01	0.434
Error	72	0.300468	0.004173		
Total	95	0.397137			

Table S5. ANOVA for Box-Cox transformed DMSP concentrations in *U. fenestrata* within a meter of DMS emitters on day 11 of the field experiment. Directions are north, east, west, and south of the emitters.

Source	df	SS	MS	F	P
Block	3	7.524	2.5080	6.42	**0.002**
Direction	1	2.385	2.3845	6.10	**0.021**
Block x Direction	3	1.871	0.6235	1.60	0.216
Error	24	9.376	0.3907		
Total	31	21.155			
